# How will UK hospitals use andexanet alfa? A review of local protocols

**DOI:** 10.1002/jha2.648

**Published:** 2023-01-24

**Authors:** Paula Glancy, David J. Sutton, Keith Gomez, Phillip L. R. Nicolson, Richard J. Buka

**Affiliations:** ^1^ Department of Clinical Haematology Newcastle Upon Tyne Hospitals NHS Foundation Trust Newcastle Upon Tyne UK; ^2^ Department of Clinical Haematology University Hospitals of North Midlands Stoke‐on‐Trent UK; ^3^ Haemophilia Centre and Thrombosis Unit Royal Free Hospital London NHS Foundation Trust London UK; ^4^ Institute of Cardiovascular Sciences University of Birmingham College of Medical and Dental Sciences Birmingham UK

1

Andexanet alfa is a reversal agent for anti‐factor Xa direct oral anticoagulants (DOACs). The clinical evidence for andexanet alfa comes from the single‐arm ANNEXA‐4 trial, and as such it is uncertain whether the therapy confers a survival benefit to patients compared to supportive management. In the United Kingdom, the National Institute for Health and Care Excellence (NICE) published its appraisal of the drug in May 2021. Indirect comparisons suggestive of benefit prompted NICE to recommend andexanet alfa as an option for reversing anticoagulation from apixaban or rivaroxaban in adults with life‐threatening bleeding from the gastrointestinal (GI) tract only, to be used in line with the inclusion and exclusion criteria of ANNEXA‐4 [1]. NICE also concluded that despite a cost per treatment of between £14,000–25,000, it is likely to be cost‐effective [2].

We hypothesized that despite a lack of gold‐standard evidence, NICE recommendations would compel NHS organisations to stock and use andexanet. We anticipated that local protocols would be heterogeneous. We aimed to identify how many centres had developed a protocol, how closely these protocols conformed to the ANNEXA‐4 inclusion and exclusion criteria, and how gatekeeping strategies would be employed to ensure judicious use.

Of 132 NHS acute care organisations in England, Wales, and Northern Ireland, we were able to obtain a contact email for a haematologist at 120. Scotland was excluded as andexanet alfa is permitted for major bleeding at any anatomical site [3]. We asked whether a protocol had been finalised, and if not whether one was in development. Where protocols were not written, we checked that this was still the case by August 2022. Protocols were scrutinised and data was captured in a Microsoft Excel (2018) spreadsheet.

Statistics were performed using GraphPad Prism version 9.00 for Windows (GraphPad Software, San Diego, California). To test the significance of differences between groups, Fisher's exact test was used, and p‐values were two‐tailed.

As of 31^st^ August 2022, responses had been received from 89 (74%) sites. Of these, 52 (58%) confirmed that a protocol was in place and 49 (55.1%) shared a copy. Two (2%) reported using another organisation's protocol and 10 (11%) had a protocol in development. Note that, 12 (11%) had no protocol and in two (2%) cases respondents explained that this was because of an active decision to not use it. The median time from NICE recommendation to protocol finalisation was 8.1 months (range 1–16), far in excess of the 3‐month target set by NICE.

All protocols specified that andexanet alfa was for GI bleeding only and 48 (98%) specified that GI bleeding must be life‐threatening. Although the criteria for major bleeding is defined in ANNEXA‐4, this appears in the supplementary information. 36 (69%) protocols did not include any criteria to define a life‐threatening bleed. Table 1 summarises the contents of protocols in relation to the NICE recommendations, and inclusion and exclusion criteria for the ANNEXA‐4 trial. No protocol included all these criteria. Interestingly, two (4%) organisations permit the use of andexanet alfa for patients treated with edoxaban despite this being outside the drug license [2, 4].

**TABLE 1:** Contents of protocols in relation to National Institute for Health and Care Excellence (NICE) recommendations and inclusion and exclusion criteria for ANNEXA‐4 trial in the context of gastrointestinal bleeding

	***n* = 49**	**%**
**NICE recommendation**		
Apixaban and rivaroxaban only	47	96
GI bleeding only	49	100
Life‐threatening or uncontrolled GI bleeding	48	98
**ANNEXA‐4 inclusion criteria**		
≥18 years of age	34	69
Last DOAC dose ≤18 h prior	8	16
Acute major bleeding definitions		
*Haemoglobin drop >20 g/l*	10	21
*Haemoglobin <80 g/l if no baseline is available*	8	17
*Low cardiac output*	4	9
*Mental confusion*	4	9
*Poor skin perfusion*	4	9
*Severe hypotension*	9	19
**ANNEXA‐4 exclusion criteria**		
Expected survival of less than 1 month	2	4
Planned surgery within 12 h	0	0
Pregnant or breastfeeding	5	10
Sepsis or septic shock	0	0
Thrombotic event 2 weeks prior	2	4
VKA, dabigatran, PCC, rFVIIa, whole blood, plasma in past 7 days	7	14
**Other**		
Endoscopy to be arranged	19	39

ANNEXA‐4 included patients who had taken their last dose of anticoagulant less than 18 hours prior to andexanet. 25% of those treated had DOAC levels under 75ng/ml, deemed to be subtherapeutic. In our sample, only 15 (70%) protocols included any information on timing. Eight (16%), three (6%), and four (8%) recommended 18, 24, and 48‐h cut‐offs, respectively. Fourteen (29%) protocols recommended that drug levels be assessed and seven (14%) protocols state that levels can be done urgently to guide treatment decisions. It is encouraging that some laboratories can urgently measure drug levels urgently, as this can limit the use and is likely cost‐effective [5]. However, the cut‐off drug level that should be used is uncertain.

Gatekeeping, whereby consultation with a specialist is required prior to drug administration, is likely to encourage appropriate use. Forty‐one (82%) protocols stated the storage location for andexanet alfa: twenty three (47%) in an emergency drugs fridge, eight (16%) in the pharmacy, and nine (18%) in the blood bank. Storage in emergency drug fridges likely improves the speed of access to the drug but negates an opportunity for protocolised gatekeeping. In twenty one (43%) protocols, andexanet alfa required authorisation by a haematologist alone, seven (14%) by a gastroenterologist alone, and two (4%) by both gastroenterology and haematology. In three (6%), authorisation was possible by any medical professional and one (2%) required authorisation from a general medical consultant, haematologist, and gastroenterologist. Protocols requiring authorisation by a gastroenterologist were more likely to stipulate that endoscopy should be planned: 12/19 (63%) with gastroenterology involvement compared to 7/30 (23%) when gastroenterology was not involved, p = 0.0076. This may reflect a view that andexanet alfa, which has only a three‐hour effect time [2], should be used as a bridge to the definitive management of bleeding. Centres, where there was clinically gatekeeping from either a haematologist or a gastroenterologist, were more likely to also employ stock gatekeeping – keeping the drug in either pharmacy or blood bank rather than an emergency drugs fridge (*p* = 0.031). There was no correlation between the number of inclusion/exclusion criteria contained in protocols and stock gatekeeping or clinical gatekeeping (above mean versus below mean, *p* = 1.00, *p* = 0.18 respectively). Increasing the number of specialists that need to be contacted increases the complexity of the decision‐making, decreases efficiency and may delay treatment.

Here, we have conducted a study of how individual clinical teams have interpreted NICE technology appraisal (TA‐697) for andexanet alfa. This data is important as andexanet alfa is a high‐cost drug with limited evidence of clinical benefit and evidence of harm; widespread, unrestricted use could inflict significant costs on the NHS [6]. Although most trusts have a protocol written or in development, fourteen do not ‐ indicating that andexanet alfa is not being used at all in those centres. Gatekeeping is an important consideration with decision‐making largely placed in the hands of senior haematologists or gastroenterologists. NHS organisations must strike a balance between judicious use and efficient care and should consider the unintended consequences of their approaches. The use of urgent DOAC levels should be considered where practicable as 25% of patients had subtherapeutic drug levels in ANNEXA‐4; the real‐world figure may be higher.

This study has limitations. Firstly, the response rate was 74% so we cannot provide a full picture of nationwide practice. Responder bias is plausible where centres who did not respond may be less likely to be using andexanet alfa. Also, the clinical practice may deviate from local protocols and even where a protocol is available, the drug may still not be adopted by clinicians on the ground. The ongoing HaemSTAR‐led, RAPIDO project will produce valuable real‐world data in this area [7]. It is also entirely plausible that in some centres, a thorough discussion between experienced clinicians on a case‐by‐case basis is preferred to a detailed protocol ‐ although we found no correlation between the amount of detail and gatekeeping.

In summary, local protocols for andexanet alfa in the UK are heterogeneous. Given that this is a novel agent with an uncertain risk‐benefit trade‐off and significant cost, robust protocols with appropriate gatekeeping are important to ensure consistent use that is appropriately restricted whilst avoiding delay.

## AUTHOR CONTRIBUTIONS

PG – study design, data collection, wrote the manuscript

DJS – study design, reviews of manuscript drafts

KG – reviewed data and manuscript

PLRN – reviewed data and manuscript

RJB – study design data collection, wrote the manuscript

## CONFLICT OF INTEREST

Phillip L. R. Nicolson has received research grants from Novartis, Principia, Sanofi, AstraZeneca and Rigel Pharmaceuticals. Phillip L. R. Nicolson has had honoraria from Bayer, Grifols and Takeda. David J. Sutton has received honoraria from Bayer and Bristol Myers Squibb. Richard J. Buka has received research funding from AstraZeneca. The rest of the authors have no conflict of interest.

## FUNDING INFORMATION

N/A
